# Field-to-laboratory biomechanical profiling of reactive strength index in male volleyball drop jumps

**DOI:** 10.3389/fbioe.2026.1886944

**Published:** 2026-07-29

**Authors:** Di Lu, Yueming Li, Tianyuan He, Jiaxin He, Jian Sun, Duanying Li

**Affiliations:** 1 School of Physical Education, Guangzhou Sport University, Guangzhou, Guangdong, China; 2 Sports Training Institute, Guangzhou Sport University, Guangzhou, Guangdong, China; 3 Key Laboratory of Human-Computer Intelligent Interaction for Athletic Performance and Health Promotion, Guangzhou Sport University, Guangzhou, Guangdong, China; 4 Guangdong Provincial Key Laboratory of Human Sports Performance Science, Guangzhou, Guangdong, China

**Keywords:** biomechanics, drop jump, knee joint, reactive strength index, statistical parametric mapping, volleyball

## Abstract

**Background:**

Reactive strength index (RSI) is widely used in drop-jump testing, but the score alone does not show how the landing-to-take-off phase is organized.

**Objective:**

This study examined whether RSI in male volleyball drop jumps can be interpreted more clearly by combining field-accessible performance metrics with time-continuous knee-joint biomechanical waveforms.

**Methods:**

Forty-four male volleyball participants were classified as trained athletes (n = 22) or recreational participants (n = 22). Each completed 30-cm drop jumps using force platforms and three-dimensional motion capture. Field outcomes included jump height, contact time, RSI, and countermovement depth. Laboratory outcomes included dominant-limb knee angle, moment, and power waveforms, with supplementary lower-limb peak joint and work-distribution outcomes. Discrete outcomes were analyzed with mixed models and covariate-adjusted models; waveforms were compared in Python using one-dimensional statistical parametric mapping, with supplementary body mass-normalized kinetic waveform analyses.

**Results:**

Compared with recreational participants, trained athletes had shorter contact time (adjusted β = −0.093 s, 95% CI: −0.157 to −0.030), smaller countermovement depth (adjusted β = −7.03 cm, 95% CI: −11.98 to −2.08), and higher RSI (adjusted β = 0.273 m/s, 95% CI: 0.131–0.415). Knee propulsive work was higher before anthropometric adjustment, whereas the adjusted result was statistically inconclusive. Waveform analysis showed less knee flexion across most of contact, greater knee moment during middle-to-late contact, and phase-specific knee-power differences.

**Conclusion:**

Higher RSI in trained male volleyball athletes was associated with a compact field-level landing-rebound pattern, defined by shorter contact time and smaller countermovement depth, supported by lower-limb discrete outcomes and knee-joint waveform features rather than a single biomechanical feature. Combining field-accessible metrics with lower-limb biomechanical analysis may provide a more interpretable profile of reactive strength in volleyball drop jumps.

## 1 Introduction

Drop-jump testing is a common way to assess reactive strength and stretch-shortening cycle function in athletes. In volleyball, this capacity has obvious relevance because blocking, spiking, transition play, and repeated second-jump actions all require athletes to land, control downward motion, and re-accelerate with little delay. Vertical jump height is important, but it gives only a partial view of this process. Two athletes can jump to a similar height while using very different contact times and landing depths (Tillman et al., 2004; Sheppard et al., 2008; Ziv and Lidor, 2010; Borras et al., 2011; Sattler et al., 2015; Fuchs et al., 2019; Pawlik and Mroczek, 2023; Cabarkapa et al., 2025).

The reactive strength index (RSI), typically calculated as jump height divided by contact time, is attractive in practice because it condenses a fast landing-rebound task into a single number. A higher value is often treated as evidence of better reactive strength. The practical problem is that the same number may come from different strategies: a brief and shallow contact, a longer contact offset by greater jump height, or different combinations of braking and propulsion. RSI is therefore useful for screening, but it does not by itself identify the movement strategy behind the score (Baca, 1999; Flanagan and Comyns, 2008; Turner and Jeffreys, 2010; Suchomel et al., 2016; Xu, 2023).

This limitation becomes important when trained volleyball athletes are compared with recreational players. Both groups can be familiar with volleyball movements, yet they differ in the amount and quality of repeated jump-landing exposure. Such a contrast can help explain what mechanical features accompany a higher volleyball-related RSI profile without assuming that those differences were caused by training.

Most drop-jump studies report discrete variables such as jump height, contact time, peak force, joint moment, or joint power. These outcomes are easy to communicate, but they reduce a continuous contact phase to one value. The contact phase contains a rapid shift from braking to propulsion, and joint power has different meanings across this transition: negative power reflects energy absorption, whereas positive power reflects energy generation for take-off (Viitasalo et al., 1998; Lin et al., 2008; Wang, 2011; Smith et al., 2011; Ficklin et al., 2014; Phillips and Flanagan, 2015; James et al., 2017).

The knee joint is a useful window into this profile. During landing, the knee helps manage lower-limb flexion and absorb mechanical energy. During propulsion, knee extensor output contributes to the rebound. Previous research has linked knee extensor function, knee moment, and knee power with vertical jump performance, and joint-level eccentric and concentric ratios have been used to explain stretch-shortening cycle behavior (Vanezis and Lees, 2005; Chang et al., 2015; Mersmann et al., 2017; Schons et al., 2019; Wilkosz et al., 2021; Kipp et al., 2021). Combining field metrics with knee-joint waveforms may therefore improve the interpretation of RSI and provide a field-to-laboratory biomechanical profile of the landing-to-take-off task.

From a biomechanics and human movement bioengineering perspective, a key challenge is to connect low-dimensional field-accessible performance indices with laboratory-derived joint-level mechanical information. Such integration may help translate complex biomechanical signals into interpretable athlete profiles for applied sport, rehabilitation, and human performance settings. For volleyball drop jumps, however, it remains unclear whether an RSI profile can be explained by phase-specific knee-joint waveform characteristics rather than by discrete performance metrics alone.

This study aimed to investigate whether reactive strength index (RSI) in male volleyball drop jumps can be better interpreted by integrating field-based performance metrics with time-continuous knee-joint biomechanical waveforms and supplementary lower-limb joint-level outcomes. In this study, a compact field-level landing-rebound pattern was operationally defined as shorter contact time and smaller countermovement depth interpreted together with jump height and RSI. Lower-limb context was evaluated using hip, knee, and ankle peak joint outcomes and work-distribution variables, whereas the primary time-continuous waveform analysis focused on the knee joint. We hypothesized that trained volleyball athletes would demonstrate higher RSI, shorter contact time, smaller countermovement depth, and distinct knee-joint waveform characteristics.

## 2 Materials and methods

### 2.1 Study design and participants

This cross-sectional biomechanical comparison study included 44 male volleyball participants. Participants were classified as trained volleyball athletes (n = 22) or recreational volleyball participants (n = 22) according to training background obtained during recruitment and confirmed before testing. The study was restricted to male participants because the available trained cohort represented a male competitive training environment; the findings should therefore be treated as male-specific and not generalized to female athletes without direct evidence.

Trained athletes were eligible for the athlete group if they had at least 4 years of systematic volleyball-specific training experience, had participated in at least one provincial-level or higher volleyball league, and completed structured volleyball-specific technical and tactical training three times per week, with each session lasting approximately 2 h. Thus, the current volleyball-specific training volume of the athlete group was approximately 6 h/week. All athletes also had more than 4 years of resistance-training experience, including plyometric training, strength training, and related conditioning exercises. Playing position was recorded according to the primary competitive position reported by each athlete. The athlete group included 3 outside hitters, 6 middle blockers, 4 opposites, 5 setters, and 4 liberos.

Recreational participants were eligible for the recreational group if they had no formal competitive volleyball experience and had played recreational volleyball approximately once per week during the previous year. They were not members of a structured volleyball training program and did not participate in regular competitive league training. Recreational participants reported irregular, non-systematic resistance-training exposure, but training frequency, duration, and accumulated resistance-training years were not standardized or systematically recorded. Participants were not included if they reported a current lower-limb injury or pain that limited maximal jumping, were unable to complete the familiarization and drop-jump protocol safely, or produced signals that could not be processed reliably. All participants were familiar with the drop-jump task before testing and completed the same task familiarization procedure.

The Institutional Review Board of Guangzhou Sport University approved the study (approval No. 2025LCLL-001). All participants gave written informed consent after being informed of the procedures, potential risks, and right to withdraw. The protocol was prospectively registered in the Chinese Clinical Trial Registry (ChiCTR2500105182). The sensitivity power analysis was performed in G*Power 3.1 using a two-tailed independent-samples group comparison framework. With 22 participants per group, 80% power, and α = 0.05, the available sample could detect a between-group effect of approximately d = 0.86 (Faul et al., 2007). Therefore, the study was adequately powered only for large group differences, and smaller but potentially meaningful effects may have been missed.

### 2.2 Drop-jump protocol

After a standardized warm-up, participants stood on a 30-cm box, stepped forward to initiate a free fall, landed bilaterally with one foot on each force platform, and immediately performed a maximal vertical rebound jump. The warm-up followed the same sequence for all participants and progressed from general to task-specific activity: light whole-body activity, dynamic lower-limb mobility exercises, submaximal vertical jumps, and practice drop jumps. It was supervised by the testing team, performed at a light-to-moderate effort, and continued until participants could complete the drop-jump sequence safely and consistently before data collection. Hands remained on the hips to limit arm-swing contribution. Participants were instructed to jump as high as possible while minimizing ground-contact time.

The experimental design was standardized across groups by using the same 30-cm drop height, hands-on-hips condition, testing environment, verbal instructions, rest interval, event-detection criteria, and data-processing pipeline. A 90-s rest interval separated trials. Before each trial, the tester confirmed that the participant was ready to continue and reported no discomfort; additional rest was permitted when needed. This procedure was applied identically to both groups. Trials were repeated if participants used an arm swing, jumped from the box rather than stepping off, failed to land cleanly on the force platforms, lost balance, or produced signals that prevented reliable event detection. The same trial-retention rules were applied to both groups. The target for the primary analysis was three valid trials per participant. Additional valid trials retained during testing or post-processing were kept for transparency, whereas the primary analyses used the first up to three valid trials per participant. When fewer than three valid trials were available after post-processing, all usable trials were retained and a sensitivity analysis excluded participants with fewer than three valid trials. Trial availability, trial-retention rules, and quality-control checks are summarized in Supplementary Table S1. Participants contributing fewer than three valid trials to the primary analysis are listed in Supplementary Table S2 to make the handling of incomplete trial sets transparent.

### 2.3 Instrumentation and data processing

Ground-reaction forces were recorded at 1,000 Hz using two Bertec force platforms (Bertec Corporation, Columbus, OH, United States). Three-dimensional kinematics were collected at 200 Hz using a 10-camera Qualisys motion-capture system (Qualisys AB, Gothenburg, Sweden). Marker and force-platform data were processed in Visual3D (C-Motion Inc., Germantown, MD, United States). Before data collection, the motion-capture volume and force platforms were calibrated according to manufacturer procedures. Marker trajectories and ground-reaction forces were low-pass filtered with a fourth-order Butterworth filter at 15 Hz. This cutoff was selected to retain the primary movement signal during the drop-jump contact phase while attenuating high-frequency noise and was applied identically to both groups, consistent with common biomechanical filtering principles and with evidence that filtering choices can influence inverse-dynamics estimates (Robertson and Dowling, 2003; Winter, 2009; Kristianslund et al., 2012). The use of synchronized force platforms, optical stereophotogrammetry, and inverse dynamics for jump-landing biomechanics is well established, and the workflow followed published recommendations on stereophotogrammetric instrumental error, biomechanical filtering, and inverse-dynamics-based jump and landing analysis (Chiari et al., 2005; Robertson and Dowling, 2003; Winter, 2009; Kristianslund et al., 2012; Smith et al., 2011; Phillips and Flanagan, 2015). Joint moments and powers were calculated using inverse dynamics and exported in Nm and W, respectively.

Vertical ground-reaction forces from the two platforms were summed for whole-body event detection. Initial contact was defined as the first instant at which summed vertical force exceeded 10 N, and take-off as the instant at which it fell below 10 N. Jump height was calculated from flight time using the standard flight-time equation h = gt^2^/8. The lowest vertical center-of-mass position during contact separated the braking and propulsion phases. Contact time, braking time, propulsion time, and countermovement depth were derived from these events.

Because the task was bilateral, whole-body variables were calculated from summed force-platform signals. Joint-level analyses were restricted to the dominant limb recorded during testing to avoid treating two limbs from the same participant as independent observations. The dominant limb was defined as the preferred limb used to kick a ball. The knee joint was selected *a priori* as the primary time-continuous waveform focus because knee flexion, knee extensor moment, and knee power are central to controlling landing displacement and contributing to propulsion during drop-jump contact. Sagittal-plane knee angle, moment, and power waveforms were extracted for the analyzed limb. In addition, dominant-limb sagittal-plane peak flexion angle, peak moment, peak power, and peak angular velocity were extracted for the hip, knee, and ankle to provide lower-limb joint-level context. Knee propulsive work was calculated by integrating knee power over real time during the propulsion phase using the trapezoidal rule and is reported in joules. RSI was calculated at participant level as mean jump height divided by mean contact time across retained trials. Additional hip, knee, and ankle work variables and joint work-contribution percentages were calculated for transparency and are provided in Supplementary Table S4 and the Supplementary Data workbook; these supplementary lower-limb variables were used to contextualize the knee waveform findings and avoid interpreting the RSI profile from the knee joint alone.

**FIGURE 1:**
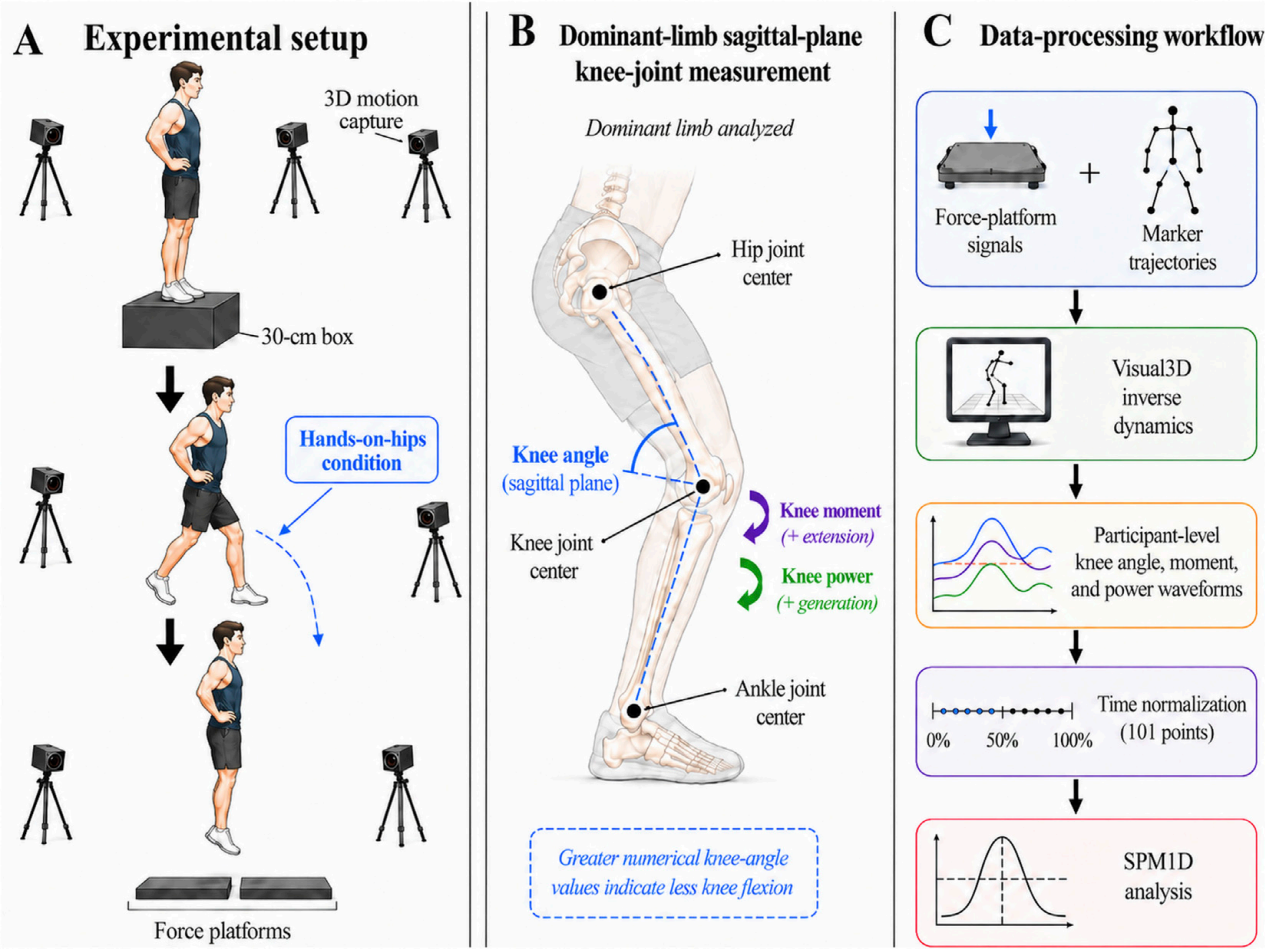
Illustration of drop-jump testing and knee-joint waveform extraction. **(A)** shows the 30-cm hands-on-hips drop-jump setup with bilateral force-platform landing and three-dimensional motion capture. **(B)** illustrates the dominant-limb sagittal-plane knee-joint measurement used for waveform extraction. **(C)** summarizes the processing workflow from force-platform and marker-trajectory data to Visual3D inverse dynamics, participant-level knee angle, moment, and power waveforms, time normalization to 101 points, and SPM1D analysis.

For waveform analysis, sagittal-plane knee angle, moment, and power were time-normalized from initial contact to take-off using 101 points. Retained trials were averaged within participant so that each participant contributed one waveform per variable. In the Visual3D model convention used here, greater knee flexion was expressed as more negative angle values; therefore, greater numerical knee-angle values indicate less knee flexion. For supplementary interpretation of waveform magnitude, group mean-difference curves and point-wise standardized effect-size curves were generated for knee angle, moment, and power and are presented in Supplementary Figure S3. The experimental setup, dominant-limb knee-joint measurement, and data-processing workflow are illustrated in Figure 1.

### 2.4 Statistical analysis

Trial-level phase and work outcomes were analyzed using linear mixed models with group as a fixed effect and participant as a random intercept. Models were repeated with age, height, and body mass as covariates. Participant-level jump height and RSI were analyzed using linear models with group as the between-subject factor, followed by adjusted models including the same covariates. Descriptive unadjusted standardized between-group effect sizes (Cohen’s d) were calculated from participant-level retained-trial means using the pooled standard deviation for major discrete outcomes to complement p-values and confidence intervals. These values were used to describe effect magnitude, whereas the model-based β estimates were used for statistical inference. Non-significant adjusted findings were interpreted as inconclusive rather than as evidence of equivalence or absence of a group difference. Trial-to-trial reliability was evaluated among participants who contributed three primary valid trials. Reliability was quantified using two-way absolute-agreement intraclass correlation coefficients for single measures [ICC(A,1)] and average measures [ICC(A,k)], together with the standard error of measurement and within-subject coefficient of variation. The reliability analysis was based on the three retained primary trials for each outcome. Reliability reporting followed recommendations for measurement error and intraclass-correlation interpretation in sports and reliability research (Atkinson and Nevill, 1998; Koo and Li, 2016).

Knee-joint angle, moment, and power waveforms were compared between groups using independent-samples one-dimensional statistical parametric mapping (SPM1D) implemented in Python (spm1d package; Pataky, 2012). Significant clusters were defined as continuous regions of the normalized contact phase where the test statistic exceeded the critical threshold, and cluster-level p-values were reported at α = 0.05. To address potential anthropometric confounding in the kinetic waveforms, supplementary analyses were conducted after normalization to body mass (knee moment: Nm/kg; knee power: W/kg). These normalized kinetic waveform analyses were intended as robustness checks because the implemented independent-samples SPM1D models did not include age, height, or body mass as covariates and did not normalize for body surface area, segment length, or limb inertial properties. Raw and body mass-normalized waveform models were evaluated using identical SPM1D procedures, and waveform findings were interpreted according to cluster timing, effect direction, and consistency across sensitivity models rather than isolated p-values. Residual waveforms were visually inspected to evaluate approximate normality and homoscedasticity. Because knee angle, moment, and power represent related but distinct biomechanical domains, multiple waveform results were interpreted cautiously and in combination with the discrete outcomes. No additional family-wise correction was applied across the three primary SPM1D waveform variables; therefore, these waveform results were interpreted as related biomechanical evidence rather than independent confirmatory tests and were evaluated together with the sensitivity analyses, body mass-normalized kinetic waveform analyses, and discrete outcomes. Sensitivity analyses excluded participants with fewer than three valid trials and used complementary point-wise models with false discovery rate correction to verify the timing and direction of waveform effects. Model diagnostics for the discrete analyses were checked by inspecting residual distributions and fitted-versus-residual plots.

## 3 Results

### 3.1 Participant characteristics

The final sample included 22 trained athletes and 22 recreational participants. The athlete group was taller and heavier than the recreational group. All trained athletes contributed three valid trials to the primary analysis, whereas eight recreational participants had fewer than three retained trials, including two participants with a single retained trial (Table 1). Supplementary Tables S1, S2 provide the trial-level transparency checks, including the eight recreational participants excluded from the sensitivity analysis because they contributed fewer than three valid trials.

**TABLE 1:** Participant characteristics, training background, and valid trial information.

Characteristic	RG	AG	AG-RG	p
Age (y)	21.40 ± 1.41	20.75 ± 0.71	−0.65	0.064
Height (cm)	179.2 ± 3.7	186.0 ± 4.9	6.7	<0.001
Body mass (kg)	71.2 ± 6.6	76.5 ± 8.9	5.3	0.032
Volleyball-specific training experience	Recreational participation only	≥4 years	NA	NA
Structured volleyball training frequency (sessions/week)	None	3	NA	NA
Structured volleyball training volume (h/week)	None	Approximately 6	NA	NA
Provincial-level or higher league experience (n)	0	22	NA	NA
Resistance-training background	Irregular, non-systematic exposure	≥4 years of structured resistance training	NA	NA
Playing position (n)	Not applicable	Outside hitter = 3; middle blocker = 6; opposite = 4; setter = 5; libero = 4	NA	NA
Available valid trials (n)	2.6 ± 0.8	5.0 ± 0.8	2.3	<0.001
Primary trials used (n)	2.5 ± 0.7	3.0 ± 0.0	0.5	0.005

Values are mean +/- SD, unless otherwise indicated; AG, trained volleyball athlete group; RG, recreational volleyball participant group. Available valid trials indicate all retained trials after post-processing; primary trials indicate trials included in the primary analysis. Volleyball-specific training experience refers to systematic volleyball-specific technical and tactical training. Structured volleyball training volume was calculated from the reported weekly training frequency and session duration. Resistance-training background in AG, included plyometric training, strength training, and related conditioning exercises. Recreational participants reported irregular, non-systematic resistance-training exposure, but detailed frequency, duration, and accumulated resistance-training years were not standardized or systematically recorded. Playing position was recorded according to each athlete’s primary competitive position.

Trial-to-trial reliability was evaluated among participants who contributed three primary valid trials (AG = 22; RG = 14). Across the primary outcomes, average-measures absolute-agreement ICCs [ICC(A,k)] ranged from 0.910 to 0.959, indicating excellent reliability for the retained trial averages. Mean within-subject coefficients of variation ranged from 4.67% for propulsion time to 8.88% for knee propulsive work. These reliability results are provided in Supplementary Table S7.

The lower trial availability in the recreational group most likely reflected greater difficulty in consistently satisfying the strict laboratory drop-jump criteria, including clean bilateral force-platform contact, balance maintenance, and reliable event detection. Importantly, the same trial-retention rules were applied to both groups, and trial availability differed before the primary-trial selection rather than because different selection rules were used for the two groups. To reduce the possibility that incomplete trial sets drove the main findings, participants with fewer than three primary valid trials were excluded in the sensitivity analysis, and reliability statistics were calculated among participants who contributed three retained primary trials. The sensitivity analysis excluded the two recreational participants who contributed only one retained trial; the persistence of the main field-level and waveform patterns indicates that these single-trial participants did not materially alter the main conclusions.

### 3.2 Discrete outcomes

**FIGURE 2:**
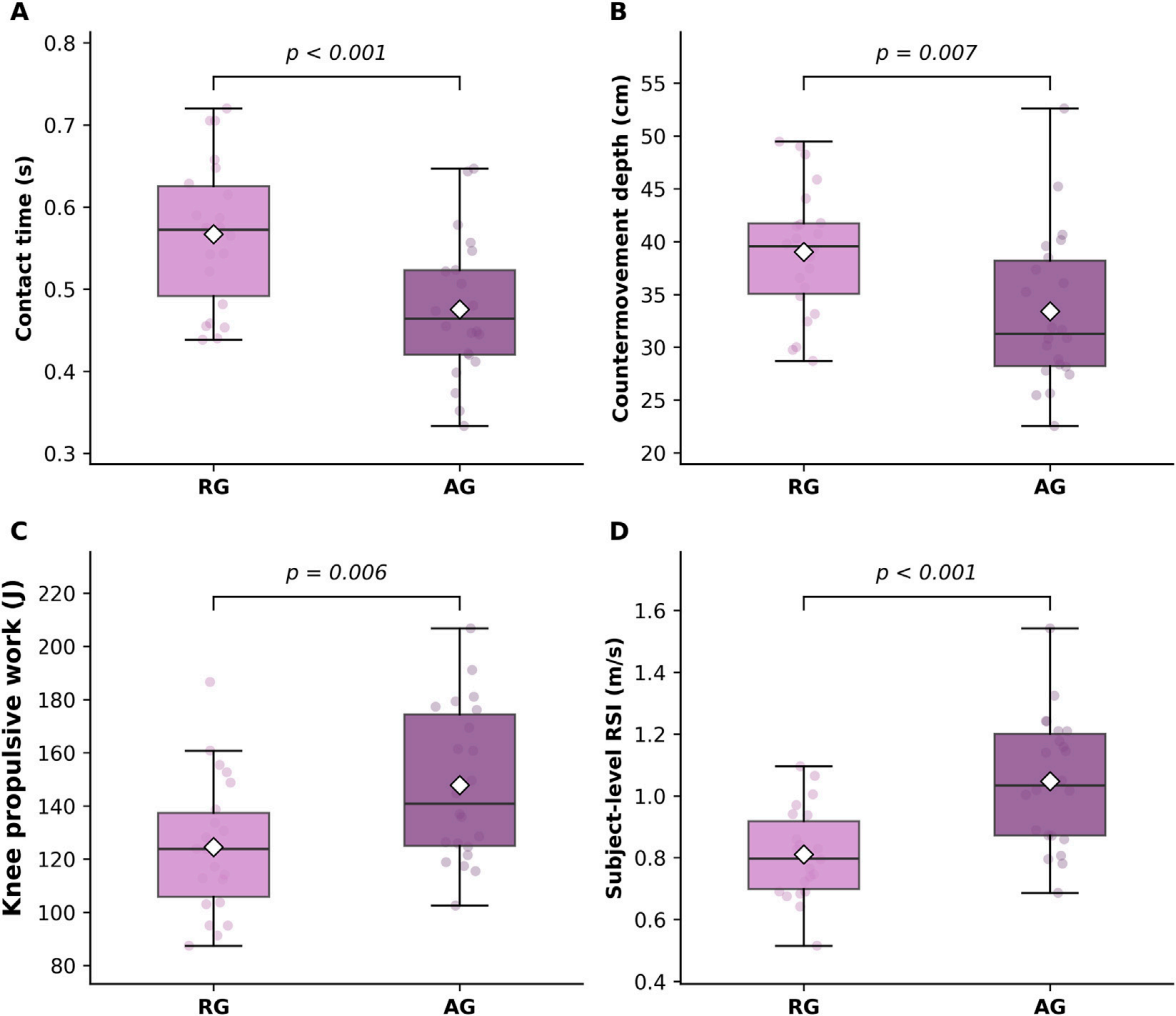
Discrete drop-jump outcomes in recreational volleyball participants (RG) and trained volleyball athletes (AG). The figure shows contact time, countermovement depth, knee propulsive work, and subject-level RSI. Boxes represent interquartile ranges, horizontal lines represent medians, diamonds represent means, and points represent individual participants. P values are from the main unadjusted group comparisons. **(A)** Contact time. **(B)** Countermovement depth. **(C)** Knee propulsive work. **(D)** Reactive strength index.

Trained athletes showed higher jump height, shorter contact time, shorter braking and propulsion times, smaller countermovement depth, and higher RSI than recreational participants (Table 2; Figure 2). Descriptive Cohen’s d values indicated moderate-to-large standardized group differences for these field-level outcomes, with the largest effects observed for RSI, braking time, and contact time. The group differences in jump height, contact time, braking time, propulsion time, countermovement depth, and RSI remained significant after adjustment for age, height, and body mass. Knee propulsive work was greater in the unadjusted model and showed a moderate-to-large descriptive standardized difference, but the adjusted estimate no longer reached statistical significance. Because the study was powered primarily to detect large group differences, this adjusted non-significant result should be interpreted as inconclusive rather than as evidence of equivalence or absence of a group difference. Thus, the most stable between-group differences were the field-level timing, displacement, jump height, and RSI outcomes. After excluding participants with fewer than three valid trials, the sensitivity sample included 36 participants (AG = 22; RG = 14), and the main field-level pattern remained broadly consistent, supporting the robustness of the primary findings (Supplementary Table S3).

**TABLE 2:** Discrete field, reactive-strength, and phase-specific outcomes.

Outcome	RG mean ± SD	AG mean ± SD	Cohen’s d	Main β (95% CI)	Main p	Adjusted β (95% CI)	Adjusted p
Jump height (cm)	44.93 ± 4.29	48.31 ± 4.80	0.74	3.38 (0.61, 6.15)	0.018	4.61 (1.29, 7.92)	0.008
Contact time (s)	0.567 ± 0.088	0.475 ± 0.085	−1.06	−0.090 (−0.140, −0.041)	<0.001	−0.093 (−0.157, −0.030)	0.006
Braking time (s)	0.275 ± 0.053	0.221 ± 0.047	−1.08	−0.053 (−0.082, −0.024)	<0.001	−0.057 (−0.094, −0.020)	0.004
Propulsion time (s)	0.292 ± 0.037	0.255 ± 0.040	−0.96	−0.037 (−0.059, −0.015)	0.002	−0.037 (−0.065, −0.008)	0.016
CM depth (cm)	39.04 ± 6.10	33.41 ± 7.26	−0.84	−5.59 (−9.48, −1.69)	0.007	−7.03 (−11.98, −2.08)	0.008
Knee propulsive work (J)	124.59 ± 25.26	147.81 ± 28.96	0.85	23.16 (7.41, 38.91)	0.006	17.02 (−1.72, 35.76)	0.082
Subject-level RSI (m/s)	0.811 ± 0.146	1.047 ± 0.212	1.30	0.237 (0.126, 0.347)	<0.001	0.273 (0.131, 0.415)	<0.001

CM, countermovement. Main β represents the unadjusted fixed group effect for AG relative to RG. Adjusted β represents the fixed group effect after adjustment for age, height, and body mass. Cohen’s d represents the unadjusted standardized mean difference for AG relative to RG, calculated from participant-level retained-trial means using the pooled standard deviation; positive values indicate higher values in AG and negative values indicate lower values in AG. Phase and work outcomes were analyzed using linear mixed models with participant as a random effect. Jump height and RSI were analyzed at the participant level. Knee propulsive work was obtained by integrating knee power over real time during the propulsive phase and is reported in joules.

Additional lower-limb peak joint outcomes in the Supplementary Data workbook provided further context for the field-level pattern. Trained athletes showed smaller peak flexion angles at the hip, knee, and ankle (Hedges’ g = −0.96, −1.00, and −0.80, respectively; all p ≤ 0.010). Kinetic differences were most evident at the knee, with greater peak knee moment (Hedges’ g = 1.49, p < 0.001) and peak knee power (Hedges’ g = 1.13, p < 0.001), whereas hip and ankle peak kinetic differences were smaller or not statistically significant. These results indicate that the compact field-level pattern was accompanied by multi-joint sagittal-plane postural differences, while the strongest kinetic evidence was concentrated at the knee. Exploratory multi-joint work outcomes are summarized in Supplementary Table S4. In both groups, knee work accounted for the largest share of braking work absorption (RG: 55.5%; AG: 54.8%), whereas propulsion work was more distributed across the hip, knee, and ankle. Descriptively, AG showed a slightly higher knee propulsion contribution (45.9% vs. 42.2%), a lower hip propulsion contribution (25.9% vs. 29.6%), and a similar ankle propulsion contribution (28.2% in both groups). These variables provided lower-limb context for interpreting the knee waveform results and helped avoid interpreting RSI from the knee joint alone.

### 3.3 Knee-joint waveforms

**FIGURE 3:**
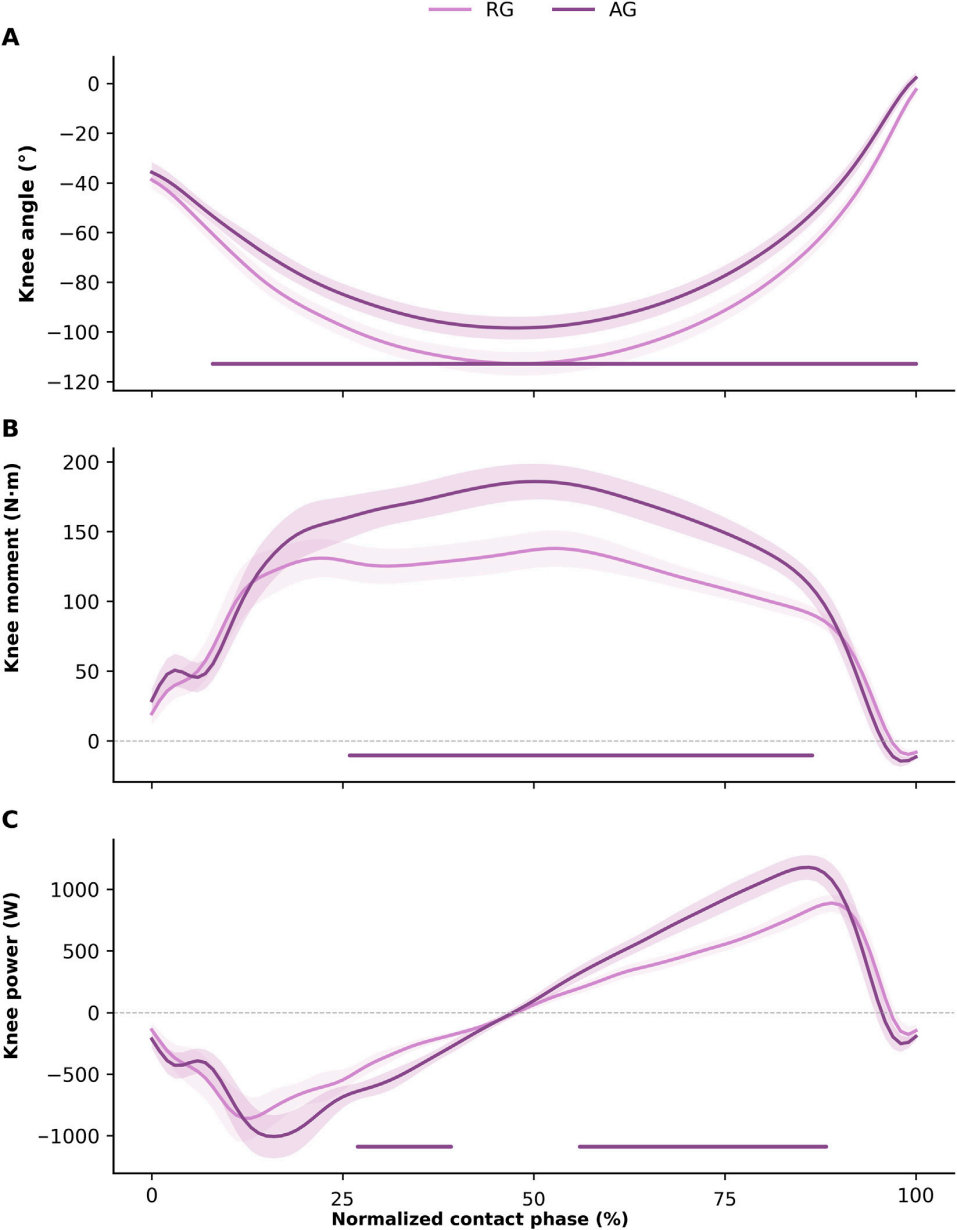
Knee-joint waveforms across the normalized drop-jump contact phase. Solid lines represent recreational volleyball participants and dashed lines represent trained volleyball athletes; shaded regions represent 95% confidence intervals. Purple horizontal bars indicate significant clusters identified using SPM1D. Knee moment and knee power are reported as N m and W, respectively; the x-axis represents the normalized contact phase. **(A)** Knee angle. **(B)** Knee moment. **(C)** Knee power.

SPM1D showed clear group differences across the normalized contact phase (Table 3; Figure 3). Knee angle differed from 8.0% to 100.0% of contact, indicating less knee flexion in trained athletes. Knee moment was greater in the athlete group from 25.9% to 86.4% of contact. Knee power showed two clusters: trained athletes had lower knee power from 27.0% to 39.2%, indicating greater negative power absorption during braking, and greater knee power from 56.0% to 88.2%, indicating greater positive power generation during propulsion.

**TABLE 3:** Main-analysis SPM1D significant clusters for knee-joint waveforms.

Variable	Interval (% contact)	Direction	Peak t	Cluster p	Interpretation
Knee angle	8.0–100.0	AG > RG	5.06	<0.001	AG showed less knee flexion
Knee moment (N·m)	25.9–86.4	AG > RG	6.20	<0.001	AG showed greater knee moment
Knee power (W)	27.0–39.2	AG < RG	−4.96	<0.001	AG showed greater negative power absorption
Knee power (W)	56.0–88.2	AG > RG	6.52	<0.001	AG showed greater positive power

Intervals indicate portions of the normalized contact phase showing significant SPM1D clusters. Knee moment is expressed in Nm; knee power is expressed in W. for knee angle, greater numerical values reflected less knee flexion because flexion was represented by more negative values in the model convention. Supplementary body mass-normalized kinetic waveform clusters are reported in Supplementary Table S6.

The SPM1D sensitivity analysis excluding participants with fewer than three valid trials retained the main waveform pattern. Significant clusters were observed for knee angle from 3.7% to 100.0% of contact, knee moment from 28.9% to 85.0% of contact, and knee power during early braking and propulsion. A small terminal knee-moment cluster was also detected from 99.4% to 100.0% of contact (Supplementary Figure S1). Complementary point-wise models with false discovery rate correction showed broadly similar timing and direction of waveform effects (Supplementary Figure S2). The corresponding primary-analysis and sensitivity-analysis cluster summaries are provided in Supplementary Table S5, and the full SPM1D t-value series are provided in the Supplementary Data workbook.

Supplementary SPM1D analyses using body mass-normalized knee-joint kinetic waveforms showed temporal patterns broadly consistent with the raw waveform analyses (Supplementary Table S6). Body mass-normalized knee moment remained greater in trained athletes from 27.86% to 85.04% of contact (cluster p < 0.001). Body mass-normalized knee power showed two significant clusters: trained athletes had lower knee power from 30.67% to 38.63% of contact (cluster p < 0.001), indicating greater negative knee power during braking, and greater knee power from 59.57% to 87.08% of contact (cluster p < 0.001), indicating greater positive knee power during propulsion. In the sensitivity analysis, body mass-normalized knee moment remained significant from 43.41% to 49.22% and from 55.05% to 83.49% of contact, and the positive knee-power cluster during propulsion was retained from 67.44% to 83.77% of contact. The early negative knee-power cluster observed in the main body mass-normalized analysis was not retained in the sensitivity analysis and should therefore be interpreted more cautiously.

## 4 Discussion

This study shows that a higher RSI in male volleyball drop jumps is not merely a better field score. In trained athletes, it was accompanied by less time on the ground, a smaller countermovement depth, smaller peak flexion angles across the hip, knee, and ankle, less knee flexion across most of contact, greater knee moment through the middle-to-late contact phase, and clearer knee-power modulation from braking to propulsion. This supports the operational description of a compact field-level landing-rebound pattern within a lower-limb context, although the strongest time-continuous evidence came from knee-joint waveforms. Because the design was cross-sectional, these differences should be interpreted as characteristics associated with training status, not as proof that training caused the observed mechanics.

The strongest practical message is that RSI should be read together with the variables that create it. In this sample, trained athletes did not reach higher RSI by using a deeper or slower countermovement. Instead, they achieved the task with a shorter, shallower contact while also showing greater jump height. This pattern is consistent with prior work showing the importance of contact-time regulation, jump height, and stretch-shortening cycle function in jumping tasks and volleyball performance (Flanagan and Comyns, 2008; Sheppard et al., 2008; Borras et al., 2011; Suchomel et al., 2016; Pawlik and Mroczek, 2023). For coaches and applied biomechanists, RSI remains a useful first look, but contact time, jump height, and countermovement depth help identify whether the score reflects a fast-shallow strategy or a more displacement-based rebound.

The lower-limb peak-angle and knee-angle waveform results give mechanical context to this field pattern. Trained athletes showed smaller peak flexion angles at the hip, knee, and ankle, and they maintained less knee flexion for nearly the entire contact phase, matching their smaller countermovement depth. This supports a lower-limb compactness interpretation during the controlled drop-jump task, while recognizing that the time-continuous waveform evidence was centered on the knee joint. Such a profile may help limit downward displacement during this task, but it should not be treated as universally superior. In other tasks or fatigue states, too little flexion may reduce impact attenuation or concentrate loading. The interpretation should remain task-specific.

The knee moment and power findings add a second layer. Trained athletes showed greater knee moment over the middle-to-late part of contact, when the task shifts from controlling descent to preparing take-off. Knee propulsive work was higher before adjustment but was attenuated after accounting for age, height, and body mass. Given the moderate-to-large descriptive effect size and the limited power to detect smaller adjusted effects, this result should be viewed as supportive but statistically inconclusive rather than as evidence that the groups were equivalent in knee propulsive work. The waveform findings are more informative because they show when the group difference occurred rather than only whether a single summary value differed.

The knee-power waveform is particularly useful for understanding the landing-to-take-off transition. Trained athletes showed greater negative knee power during braking and greater positive knee power during propulsion in the raw waveform analysis. The group difference was therefore not a simple increase in power across the whole movement but a phase-dependent knee-joint waveform pattern. However, because the early braking-phase difference was not retained in the body mass-normalized sensitivity analysis, the braking-phase interpretation should be treated cautiously. The propulsive-phase knee-power difference was more robust across the raw, body mass-normalized, and sensitivity analyses.

The additional hip and ankle results reinforce that the drop jump should be interpreted as a multi-joint task. The lower-limb peak-angle results showed a broader sagittal-plane postural pattern, and the exploratory work-distribution data showed that braking and propulsion involved contributions from the hip, knee, and ankle. Therefore, the present conclusions are framed as a lower-limb landing-rebound profile supported by multi-joint discrete and work-distribution outcomes and centered on knee-joint waveform evidence, rather than as a complete causal account of whole-limb coordination or inter-joint energy transfer.

The trial-to-trial reliability analysis also helps address the unequal trial-retention issue. Among participants who contributed three primary valid trials, ICC(A,k) values for the primary outcomes were excellent, supporting the stability of the retained trial averages used for participant-level interpretation. This reliability evidence complements, rather than replaces, the sensitivity analysis excluding participants with fewer than three valid trials.

The supplementary body mass-normalized SPM1D analyses provided additional support for the knee kinetic waveform findings while also delineating their interpretive boundaries. After normalization, the mid-to-late contact difference in knee moment and the propulsive-phase difference in knee power were retained, suggesting that these waveform patterns were not attributable solely to between-group differences in body mass. In contrast, the early braking-phase negative knee-power cluster was not preserved in the normalized sensitivity analysis, indicating that this finding is less robust than the propulsive-phase power difference. It should also be noted that body mass normalization cannot fully account for residual anthropometric and training-related confounding, including differences in segment length, limb inertial properties, muscle mass, tendon properties, training age, or strength level. Therefore, the kinetic waveform results should be interpreted as group-associated knee-joint biomechanical profiles rather than direct evidence that training status alone caused the observed mechanical differences.

From a human movement bioengineering perspective, the present findings show how field-accessible performance metrics can be linked to time-continuous joint-level mechanical signals. This field-to-laboratory integration may help translate complex biomechanical waveforms into interpretable athlete profiles, particularly when repeated laboratory testing is not feasible in routine sport settings. The approach is not meant to replace simple monitoring with laboratory testing; rather, waveform analysis can be used periodically when individualized biomechanical profiling is needed. Providing the anonymized supplementary tables and data workbook also allows readers to inspect trial retention, sensitivity analyses, and exploratory joint-work outputs beyond the condensed main-text tables, improving the reproducibility and auditability of the biomechanical interpretation.

### 4.1 Practical implications

- RSI is most useful when interpreted with jump height, contact time, and countermovement depth rather than as an isolated score.
- A long contact time combined with large countermovement depth may indicate a slower, more displacement-based landing-rebound strategy.
- A short contact time combined with limited jump height may point to the need for additional assessment of propulsive capacity.
- Knee-joint waveform analysis can be used periodically to profile braking and propulsive strategies when laboratory resources are available.

### 4.2 Limitations and future directions

Several limitations should be considered. The groups were defined by existing training background, so causal statements about training adaptation cannot be made. The athlete group was taller and heavier. Although the discrete models included age, height, and body mass as covariates and the supplementary waveform analyses included body mass-normalized knee kinetics, these procedures cannot remove all residual confounding related to body surface area, segment length, limb inertial properties, muscle mass, tendon properties, training quality, strength level, playing position, accumulated volleyball-specific exposure, or resistance-training background. The SPM1D analyses did not include anthropometric covariates directly; therefore, the body mass-normalized waveform results should be interpreted as complementary robustness evidence rather than complete control of anthropometric confounding. Although athlete-group classification was strengthened by documenting systematic volleyball-specific training experience, current weekly training volume, provincial-level competitive experience, playing position, and resistance-training background, these descriptors should be interpreted as group-characterization variables rather than evidence that training history alone caused the observed biomechanical differences. The sample included only males, and the results should not be generalized to female volleyball athletes without direct evidence. Sex-specific differences in neuromuscular control, lower-limb strength characteristics, landing mechanics, joint-loading patterns, and volleyball positional demands may alter the relationship between RSI and lower-limb biomechanical profiles. The single 30-cm drop height and hands-on-hips condition improved control but reduced ecological similarity to volleyball actions involving arm swing, approach steps, fatigue, or repeated contacts (Zhang et al., 2021). Although the sensitivity analysis supported the main field-level and several waveform patterns, excluding participants with fewer than three valid trials reduced the recreational-group sample and should be interpreted as a robustness check rather than an independent replication. In addition, the available sample was powered primarily for large between-group effects; therefore, Type II error remains possible for smaller but potentially meaningful differences, particularly for adjusted discrete outcomes such as knee propulsive work. In addition, failed-trial reasons were not systematically coded by category; therefore, the exact contribution of specific factors such as foot placement, balance loss, or event-detection failure to the lower recreational-group trial retention could not be quantified directly. Finally, although lower-limb discrete joint outcomes were used to provide multi-joint context, the time-continuous waveform analysis focused on the dominant-limb knee joint. Future studies should include bilateral limb behavior, hip-ankle waveform coordination, inter-joint energy redistribution, muscle-tendon behavior, female athletes, and longitudinal designs.

## 5 Conclusion

In male volleyball drop jumps, trained athletes showed higher RSI together with greater jump height, shorter contact time, smaller countermovement depth, lower-limb sagittal-plane postural differences, and distinct knee-joint waveform characteristics. The body mass-normalized waveform analyses supported mid-to-late contact knee-moment differences and a robust propulsive-phase knee-power difference, whereas the braking-phase power difference was less stable in sensitivity analysis. Overall, higher RSI was associated with a compact field-level landing-rebound pattern, operationally defined by shorter contact time and smaller countermovement depth, and a lower-limb biomechanical profile supported by hip-knee-ankle discrete outcomes and knee-joint waveform characteristics, while residual anthropometric and training-related confounding should still be considered.

## Data Availability

The raw data supporting the conclusions of this article will be made available by the authors, without undue reservation.
