# Supplementary material for: Field-to-laboratory biomechanical profiling of reactive strength index in male volleyball drop jumps
Source: Front Bioeng Biotechnol. 2026 Jul 29;14:1886944. doi: 10.3389/fbioe.2026.1886944 (PMC13462052; doi:10.3389/fbioe.2026.1886944)
Supplement: Supplementary file 1 [file Supplementaryfile1.docx]

**Supplementary material**

This supplementary material provides additional data transparency tables, sensitivity-analysis details, body mass-normalized waveform results, trial-to-trial reliability statistics, and supplementary figures supporting the manuscript submitted to Frontiers in Bioengineering and Biotechnology, Section: Biomechanics. Participant names have been removed from all supplementary tables; subject labels are retained only for traceability across datasets.

**Supplementary Table 1. Dataset structure, trial retention, and quality-control overview.**

| **Dataset component** | **AG** | **RG** | **Total** | **Notes** |
| --- | --- | --- | --- | --- |
| Participants included in primary analyses | 22 | 22 | 44 | All included participants |
| Participants retained in sensitivity analyses | 22 | 14 | 36 | Participants with ≥3 primary trials |
| Participants excluded from sensitivity analyses | 0 | 8 | 8 | Participants with fewer than 3 primary trials |
| All available valid trial rows in phase/work dataset | 109 | 58 | 167 | All valid trials before primary-trial selection |
| Primary trial rows used in main phase/work analyses | 66 | 56 | 122 | First up to 3 valid trials per participant |
| Primary trial rows retained in sensitivity analyses | 66 | 42 | 108 | After excluding participants with <3 primary trials |
| Participants with a single primary trial | 0 | 2 | 2 | Subgroup retained in primary but excluded from sensitivity analyses |
| Trial rows passing phase-time QC | 109/109 | 58/58 | 167/167 | QC was evaluated on all valid trial rows |
| Trial rows passing COM-depth QC | 109/109 | 58/58 | 167/167 | QC was evaluated on all valid trial rows |
| Trial rows passing work-sign QC | 107/109 | 58/58 | 165/167 | QC was evaluated on all valid trial rows |

Note. AG = trained volleyball athlete group; RG = recreational volleyball participant group; QC = quality control; COM = center of mass.

**Supplementary Table 2. Participants with fewer than three primary valid trials and their retained primary-trial means.**

| **Subject ID** | **Group** | **Available trials** | **Primary trials** | **Primary CASTs** | **Jump height (cm)** | **Contact time (s)** | **Braking time (s)** | **Propulsion time (s)** | **CM depth (cm)** | **Knee propulsive work (J)** | **RSI (m/s)** |
| --- | --- | --- | --- | --- | --- | --- | --- | --- | --- | --- | --- |
| S33 | RG | 2 | 2 | 1,2 | 51.02 | 0.615 | 0.312 | 0.302 | 40.75 | 95.04 | 0.830 |
| S34 | RG | 2 | 2 | 4,5 | 51.81 | 0.647 | 0.333 | 0.315 | 44.08 | 138.67 | 0.800 |
| S40 | RG | 2 | 2 | 1,6 | 37.09 | 0.542 | 0.253 | 0.290 | 41.64 | 125.41 | 0.684 |
| S43 | RG | 2 | 2 | 5,7 | 47.52 | 0.657 | 0.340 | 0.318 | 35.68 | 117.15 | 0.723 |
| S44 | RG | 2 | 2 | 3,6 | 39.84 | 0.590 | 0.278 | 0.312 | 39.80 | 123.90 | 0.675 |
| S45 | RG | 1 | 1 | 4 | 37.09 | 0.720 | 0.350 | 0.370 | 45.87 | 152.69 | 0.515 |
| S51 | RG | 1 | 1 | 1 | 49.45 | 0.575 | 0.280 | 0.295 | 40.31 | 103.08 | 0.860 |
| S56 | RG | 2 | 2 | 1,3 | 41.25 | 0.440 | 0.207 | 0.233 | 32.45 | 91.30 | 0.938 |

Note. These participants were retained in the primary analysis according to the prespecified rule but excluded from sensitivity analyses. Participant names were removed.

**Supplementary Table 3. Sensitivity analysis excluding participants with fewer than three primary valid trials.**

| **Outcome** | **RG mean ± SD** | **AG mean ± SD** | **Main β (95% CI)** | **Main p** | **Adjusted β (95% CI)** | **Adjusted p** |
| --- | --- | --- | --- | --- | --- | --- |
| Jump height (cm) | 45.25 ± 2.93 | 48.31 ± 4.80 | 3.07 (0.16, 5.97) | 0.039 | 4.10 (0.85, 7.35) | 0.015 |
| Contact time (s) | 0.549 ± 0.097 | 0.475 ± 0.086 | -0.074 (-0.130, -0.018) | 0.010 | -0.080 (-0.147, -0.013) | 0.019 |
| Braking time (s) | 0.264 ± 0.062 | 0.221 ± 0.048 | -0.043 (-0.076, -0.011) | 0.009 | -0.050 (-0.088, -0.011) | 0.012 |
| Propulsion time (s) | 0.285 ± 0.038 | 0.255 ± 0.041 | -0.030 (-0.055, -0.005) | 0.019 | -0.031 (-0.061, -0.000) | 0.047 |
| CM depth (cm) | 38.45 ± 7.45 | 33.41 ± 7.50 | -5.04 (-9.71, -0.38) | 0.034 | -6.33 (-11.92, -0.73) | 0.027 |
| Knee propulsive work (J) | 128.12 ± 28.29 | 147.81 ± 30.96 | 19.69 (1.23, 38.16) | 0.037 | 14.83 (-5.31, 34.97) | 0.149 |
| Subject-level RSI (m/s) | 0.844 ± 0.147 | 1.047 ± 0.212 | 0.204 (0.072, 0.336) | 0.004 | 0.242 (0.083, 0.402) | 0.004 |

Note. Trial-level outcomes were analyzed with participant-level random intercepts. Jump height and RSI were analyzed at the subject level. Adjusted models included age, height, and body mass as covariates. β represents AG relative to RG.

**Supplementary Table 4. Additional phase-specific joint work and work-contribution outcomes.**

| **Supplementary variable** | **RG mean ± SD** | **AG mean ± SD** | **Source/notes** |
| --- | --- | --- | --- |
| Hip braking work (J) | -69.86 ± 32.24 | -64.04 ± 21.34 | Subject-level means from primary retained trials |
| Knee braking work (J) | -117.08 ± 34.20 | -122.84 ± 37.13 | Subject-level means from primary retained trials |
| Ankle braking work (J) | -26.30 ± 13.24 | -36.23 ± 17.28 | Subject-level means from primary retained trials |
| Total braking work absorption (J) | 213.24 ± 57.12 | 223.12 ± 59.19 | Subject-level means from primary retained trials |
| Hip braking work contribution (%) | 32.3 ± 10.4 | 28.8 ± 6.8 | Subject-level means from primary retained trials |
| Knee braking work contribution (%) | 55.5 ± 9.8 | 54.8 ± 6.9 | Subject-level means from primary retained trials |
| Ankle braking work contribution (%) | 12.2 ± 4.9 | 16.4 ± 6.0 | Subject-level means from primary retained trials |
| Hip propulsion work (J) | 88.09 ± 23.50 | 83.42 ± 25.88 | Subject-level means from primary retained trials |
| Knee propulsion work (J) | 124.59 ± 25.26 | 147.81 ± 28.96 | Subject-level means from primary retained trials |
| Ankle propulsion work (J) | 83.02 ± 15.35 | 89.82 ± 12.57 | Subject-level means from primary retained trials |
| Total propulsion work (J) | 295.71 ± 40.73 | 321.05 ± 38.27 | Subject-level means from primary retained trials |
| Hip propulsion work contribution (%) | 29.6 ± 5.9 | 25.9 ± 6.8 | Subject-level means from primary retained trials |
| Knee propulsion work contribution (%) | 42.2 ± 6.5 | 45.9 ± 5.9 | Subject-level means from primary retained trials |
| Ankle propulsion work contribution (%) | 28.2 ± 4.6 | 28.2 ± 4.1 | Subject-level means from primary retained trials |

Note. Values are subject-level means derived from the primary retained trials. Braking work is reported as negative joint work for individual joints; total braking work is presented as absolute absorption. Ratio variables are expressed as percentages of the corresponding phase total.

**Supplementary Table 5. Main and sensitivity SPM1D cluster results for knee-joint waveforms.**

| **Analysis** | **Waveform** | **Cluster** | **Interval (% contact)** | **Direction** | **Mean t** | **Cluster p** |
| --- | --- | --- | --- | --- | --- | --- |
| Main SPM1D analysis | Knee Angle | 1 | 8.0%-100.0% | AG > RG | 4.32 | p < 0.001 |
| Main SPM1D analysis | Knee Moment | 1 | 25.9%-86.4% | AG > RG | 5.21 | p < 0.001 |
| Main SPM1D analysis | Knee Power | 1 | 27.0%-39.2% | AG < RG | -4.05 | p < 0.001 |
| Main SPM1D analysis | Knee Power | 2 | 56.0%-88.2% | AG > RG | 5.26 | p < 0.001 |
| Sensitivity SPM1D analysis | Knee Angle | 1 | 3.7%-100.0% | AG > RG | 4.08 | p < 0.001 |
| Sensitivity SPM1D analysis | Knee Moment | 1 | 28.9%-85.0% | AG > RG | 4.19 | p < 0.001 |
| Sensitivity SPM1D analysis | Knee Moment | 2 | 99.4%-100.0% | AG < RG | -3.21 | p = 0.050 |
| Sensitivity SPM1D analysis | Knee Power | 1 | 27.3%-30.1% | AG < RG | -3.50 | p = 0.028 |
| Sensitivity SPM1D analysis | Knee Power | 2 | 32.9%-36.5% | AG < RG | -3.48 | p = 0.020 |
| Sensitivity SPM1D analysis | Knee Power | 3 | 63.9%-86.9% | AG > RG | 4.36 | p < 0.001 |

Note. Waveform analyses were conducted in Python using the spm1d package. The sensitivity analysis excluded participants with fewer than three primary valid trials.

**Supplementary Table 6. Body mass-normalized SPM1D cluster results for knee-joint kinetic waveforms.**

| **Analysis** | **Waveform** | **Cluster** | **Interval (% contact)** | **Direction** | **Peak t** | **Cluster p** | **Interpretation** |
| --- | --- | --- | --- | --- | --- | --- | --- |
| Main body mass-normalized SPM1D | Knee moment (N·m/kg) | 1 | 27.86%-85.04% | AG > RG | 5.48 | p < 0.001 | AG showed greater body mass-normalized knee moment |
| Main body mass-normalized SPM1D | Knee power (W/kg) | 1 | 30.67%-38.63% | AG < RG | -4.44 | p < 0.001 | AG showed greater negative knee power during braking |
| Main body mass-normalized SPM1D | Knee power (W/kg) | 2 | 59.57%-87.08% | AG > RG | 5.53 | p < 0.001 | AG showed greater positive knee power during propulsion |
| Sensitivity body mass-normalized SPM1D | Knee moment (N·m/kg) | 1 | 43.41%-49.22% | AG > RG | 3.26 | p = 0.025 | AG showed greater body mass-normalized knee moment |
| Sensitivity body mass-normalized SPM1D | Knee moment (N·m/kg) | 2 | 55.05%-83.49% | AG > RG | 4.59 | p < 0.001 | AG showed greater body mass-normalized knee moment |
| Sensitivity body mass-normalized SPM1D | Knee power (W/kg) | 1 | 67.44%-83.77% | AG > RG | 4.11 | p < 0.001 | AG showed greater positive knee power during propulsion |

Note. Body mass-normalized analyses were performed for knee kinetic waveforms only. Knee moment was expressed as N·m/kg and knee power as W/kg. The sensitivity analysis excluded participants with fewer than three primary valid trials. The braking-phase knee-power cluster observed in the main normalized analysis was not retained in the sensitivity analysis and should therefore be interpreted cautiously.

**Supplementary Table 7. Trial-to-trial reliability statistics for primary outcomes.**

| **Outcome** | **n** | **Trials** | **ICC(A,1)** | **ICC(A,k)** | **SEM** | **CV%, mean ± SD** | **Rating** |
| --- | --- | --- | --- | --- | --- | --- | --- |
| Braking time (s) | 36 | 3 | 0.771 | 0.91 | 0.028 | 8.60 ± 6.83 | Excellent |
| Contact time (s) | 36 | 3 | 0.839 | 0.94 | 0.039 | 5.94 ± 4.69 | Excellent |
| Countermovement depth (cm) | 36 | 3 | 0.845 | 0.942 | 3.116 | 7.64 ± 4.50 | Excellent |
| Knee propulsive work (J) | 36 | 3 | 0.818 | 0.931 | 13.48 | 8.88 ± 4.87 | Excellent |
| Propulsion time (s) | 36 | 3 | 0.886 | 0.959 | 0.014 | 4.67 ± 2.96 | Excellent |

Note. Reliability was calculated among participants who contributed three primary valid trials (AG = 22; RG = 14). ICC(A,1) = two-way absolute-agreement single-measure intraclass correlation coefficient; ICC(A,k) = two-way absolute-agreement average-measures intraclass correlation coefficient; SEM = standard error of measurement; CV = within-subject coefficient of variation. Interpretation is based on ICC(A,k).

**Supplementary Figure 1. SPM1D sensitivity analysis excluding participants with fewer than three valid trials. Purple shaded areas and horizontal bars indicate significant SPM1D clusters across the normalized contact phase.**

**Supplementary Figure 2. Complementary point-wise analysis with false discovery rate correction. Purple shaded areas indicate significant portions of the normalized contact phase after correction.**

**Supplementary Figure 3. Mean-difference and point-wise standardized effect-size curves for knee-joint waveforms. Left panels show the group mean difference (AG − RG) across the normalized contact phase for knee angle, knee moment, and knee power. Right panels show the corresponding point-wise standardized effect-size curves expressed as Cohen's d. Horizontal dashed lines indicate zero difference, and purple shaded regions indicate the main SPM1D significant intervals.**
